# Davydov-Moore vaginoplasty in Mayer-Rokitansky-Küster-Hauser syndrome: sexual and surgical outcomes

**DOI:** 10.1007/s00404-024-07830-6

**Published:** 2024-11-13

**Authors:** Magdalena Piróg, Magdalena Bednarczyk, Katarzyna Barabasz, Olga Kacalska-Janssen, Robert Jach

**Affiliations:** Gynecological Endocrinology Department, Jagiellonian University Medical College, ul. Kopernika 23, 31-501, Krakow, Poland

**Keywords:** Case report, Davydov-Moore vaginoplasty, MRKH syndrome, Primary amenorrhea, Vaginal agenesis

## Abstract

**Purpose:**

The aim of this study was to compare surgical and sexual outcomes after Davidov-Moore vaginoplasty in women with Mayer-Rokitansky-Küster-Hauser syndrome (MRKH).

**Methods:**

In the case-series study, we described seven women, at a median age of 22.6 ± and BMI 22.8 ± 2.3 kg/m^2^. We measured peri- and postsurgical parameters, including surgery-related neovaginal length and sexual initiation time. Sexual outcomes were measured using the Female Sexual Function Index (FSFI) before and 6 months after vaginoplasty.

**Results:**

All surgical procedures were performed successfully, with one minor perioperative bleeding. The mean time of vaginoplasty was 82.1 min and the mean duration of hospitalization was six days. After a 6-month follow-up, vaginal length was 8.1-times longer than before surgery (10 vs. 81 mm). The time from the surgery to the initiation of vaginal intercourse was between 17 to 22 weeks. The mean FSFI score indicated good results, with no women below 23 score, and was 4.3- times higher when compared with the pre-surgical one (6.7 vs 29.1). Contrary to the FSFI score before surgery, the post-surgical FSFI revealed higher scores in all six different domains: desire (2.5-times), arousal (4.1-times), lubrication (3.8-times), orgasm (3.4-times), satisfaction (3.3-times) and comfort (11-times).

**Conclusion:**

Laparoscopic Davydov-Moore vaginoplasty might be considered as a safe procedure with satisfactory anatomic and sexual outcomes. It should be considered as a treatment option for the creation of neovagina in women with MRKH.

## What does this study add to the clinical work

**Table:** 

Davydov-Moore vaginoplasty should might be considered as a safe procedure with satisfactory anatomic and sexual outcomes in women with Mayer-Rokitansky-Küster-Hauser syndrome.	

## Introduction

Mayer-Rokitansky-Küster-Hauser (MRKH) syndrome is defined as a congenital absence or hypoplasia of the uterus, cervix, and vagina due to malformations in the Müllerian ducts’ development, despite the presence of typical external genitalia [1]. It affects 1 out of 5000 women with normal phenotypes and karyotypes (46, XX) [2]. Most of the cases are diagnosed between 15–17 ages due to primary amenorrhea. It is subdivided into two types, type 1 (MRKH I), related only to organs developing from the Müllerian ducts, and type 2, where additional malformations including renal and skeletal systems occur (MRKH II) [3]. The diagnostic process includes 3D ultrasonography or MRI of the pelvis along with hormonal assay [1].

The diagnosis of MRKH is not only connected to physical ailments, but also may have psychological impact related to an absence of menstruation, challenges with sexual activity along with the inability to conceive [4]. In women with MRKH and desire for sexual activity, the treatment is a vaginal reconstruction [5]. Treatment options include form non-invasive methods using dilators in case of the presence of a rudimentary vagina, to more invasive ones such as an operative creation of a neovagina or uterus transplantation, as the first true infertility treatment [6, 7]. Taking into account that no standardized treatment is maintained, the surgical approach is based on the operating surgeon’s experience [8]. One of the most common surgical procedures used to create neovagina is Davydov vaginoplasty, which includes the use of peritoneal graft [9]. It can be performed separately or combined with the procedure described by Moore et al., where vaginoplasty includes the reconstruction of the posterior vaginal canal and introitus together with the modification of the final diameter and caliber of the vagina [10].

Given the clinical significance of MRKH, we investigated the postoperative outcomes and sexual satisfaction in women with MRKH following neovaginal creation after Davydov-Moore vaginoplasty.

## Materials and methods

### Study design

In this case-series study, we enrolled seven women diagnosed with MRKH. All of them underwent Davydov-Moore vaginoplasty at the Department of Gynecological Endocrinology, University Hospital between April 2015 and Jan 2024.

### Data collection

We collected demographics, data from gynecological examination including vaginal length before the surgery, type of MRKH along with comorbidities. The data about depression, arterial hypertension, hyperprolactinemia, or insulin resistance were collected from the patient’s medical chart.

An evaluation of female sexual function was performed based on the Female Sexual Function Index (FSFI) questionnaire. Previous studies considered FSFI scores > 30 as very good functional results and scores between 23 to 29 as good results [12, 13].

### Ethical statement

The Ethics Committee at Jagiellonian University Medical College approved the study, and participants provided informed, written consent by with the Declaration of Helsinki.

### Diagnosis

Before surgery, all women underwent both pelvic and abdominal ultrasonography and MRI in the Univeristy Hospital in Krakow. The diagnostic process was initiated among women with primary amenorrhoea, vaginal agenesis, absence of the uterus, and normal external genitalia along with confirmed normal female karyotype (46, XX). The presence of additional malformations of the renal and skeletal system qualified women to MRKH II.

### Surgical treatment

The Davidov vaginoplasty was described previously [11]. Briefly, the surgical procedure was performed with the patient placed in a lithotomy position under general anesthesia, and a Foley catheter placed in the bladder before the initiation of the procedure. The modified Davydov-Moore vaginoplasty involves laparoscopic and vaginal approaches. During the laparoscopic stage, towards the apex of the tensioned the vestibule of the vagina, a site on the peritoneum was identified, around which peritoneal marginal sutures were placed in the lesser pelvis. Subsequently, a circular purse-string suture was placed along the arms of the pubic bone and promontory, below the course of the ureters and urinary bladder, achieving the upper part of the neovagina. Then, during the vaginal approach, vestibule was dissected, and a Hegar’s dilator was introduced, tensioning the peritoneum in the central portion between the marginal sutures. Under laparoscopic guidance, the peritoneum was incised above the Hegar’s dilator. The purse-string suture was tightened, shaping the peritoneal portion of the vagina. Additional single sutures were applied to approximate the edges of the upper aspect of the new vagina. A gauze dressing was applied to the newly formed vagina. Dissolvable stitches were applied to the skin.

Removal of the catheter and gauze dressing and the use of vaginal mold were scheduled 48 h after surgery. An estrogen-based vaginal cream was applied to the mold.

All seven vaginoplastic surgeries were performed by the same experienced gynecological surgeon (RJ).

### Follow-up

All patients have used vaginal mold for around 6 – 8 h each day. The follow-up including gynecological examination started before the discharge from the hospital and was carried out every month in the first 6 months after the surgery. After hospitalization, all women were asked to use a vaginal mold of increasing size (10–20) continuously for at least 12 weeks. After this period, all women were allowed to initiate sexual activity. After 6 months of follow-up the data, including the diameters and lengths were assessed and recorded along with an evaluation of female sexual function based on the FSFI questionnaire.

## Results

### Baseline characteristic

The final analysis included seven women with a median age of 22.6 ± and a BMI of 22.8 ± 2.3 kg/m^2^. The diagnosis of MRKH was made either during adolescence (*n* = 3, 42.9%) or in adulthood between 18 and 23 years (*n* = 4, 57.1%). The reason for gynecological consultation was either primary amenorrhea (*n* = 5, 71.4%) or inability of vaginal intercourse (*n* = 2, 28.6%). Except for vaginal absence/atrophy, three women had concomitant malformations including renal (*n* = 3, 100%) and skeletal systems (*n* = 1, 33.3%). None of the patients had previous surgeries. Only two women (28.6%) were without coexisting conditions, the other had at least one mentioned in Table 1.

**Table 1:** Baseline characteristic of women with Mayer-Rokitansky-Küster-Hauser syndrome (MRKH)

Parameter	
Age, yrs	22.6 ± 3.3
BMI, kg/m^2^	22.8 ± 2.3
Vaginal length before surgery, mm	10 [7.5–15]
MRKH type, *n* (%)	
I	4 (57.1)
II	3 (42.9)
Coexisting condition, n (%)	
Depression	2 (28.6)
Hypertension	1 (14.3)
Hyperprolactinemia	2 (28.6)
Insulin resistance	1 (14.3)
FSFI score	6.7 ± 1.2

*BMI* body mass index; *FSPI* Female Sexual Function Index; *MRKH* Mayer-Rokitansky-Küster-Hauser syndrome. Values are given as mean ± SD, median (interquartile range), or number (percentage)

### Peri-and postoperative outcomes

The Davidov-Moore vaginoplasty was completed successfully in all women. The mean time of surgical vaginoplasty was 1 h and twenty-two minutes (minimum 62 min and maximum 110 min). Except for one woman (14.3%) who suffered perioperative bleeding (with perioperative blood loss of around 200 ml) and needed blood transfusion, all other procedures were without major complications (Table 2). The hospitalization duration was from five to seven (in one case) days.

**Table 2:** Perioperative and postoperative outcomes after 6 months from Davidov-Moore vaginoplasty due to Mayer-Rokitansky-Küster-Hauser syndrome (MRKH)

Perioperative	
Time of surgery, min	82.1 ± 17.8
Intraoperative blood loss, mL	50 [47.5–62.5]
Major complications, n	
Internal bleeding	1
Blood transfusion	1
Time of hospitalization, days	6 ± 0.58
Postoperative	
Complications, n (%)	
Bleeding	0 (0)
Inflammation	1 (14.3)
Urine incontinence	0 (0)
Vaginal stenosis	0 (0)
Vaginal length after surgery, mm	81 [80–82.5]
Initiation time, weeks	19.7 ± 2.2
FSFI score	29.1 ± 2.2

*Initiation time* time from surgery to initiation of vaginal intercourse; *FSPI* Female Sexual Function Index. Values are given as mean ± SD, median (interquartile range), or number (percentage)

**Fig. 1:**
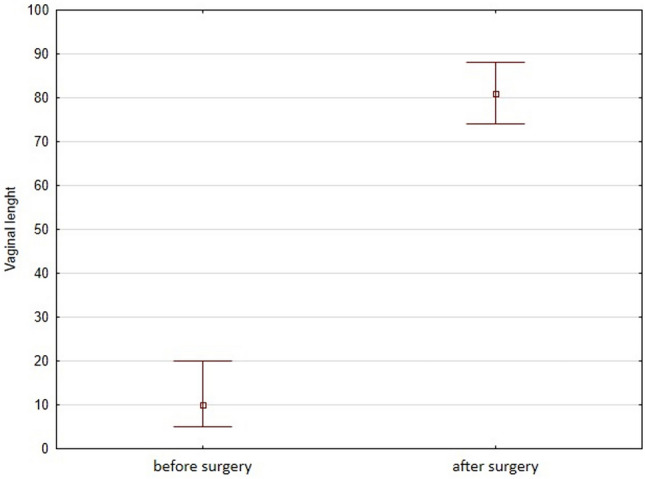
Vaginal length before and after Davidov-Moore vaginoplasty in women with Mayer-Rokitansky-Küster-Hauser syndrome

**Fig. 2:**
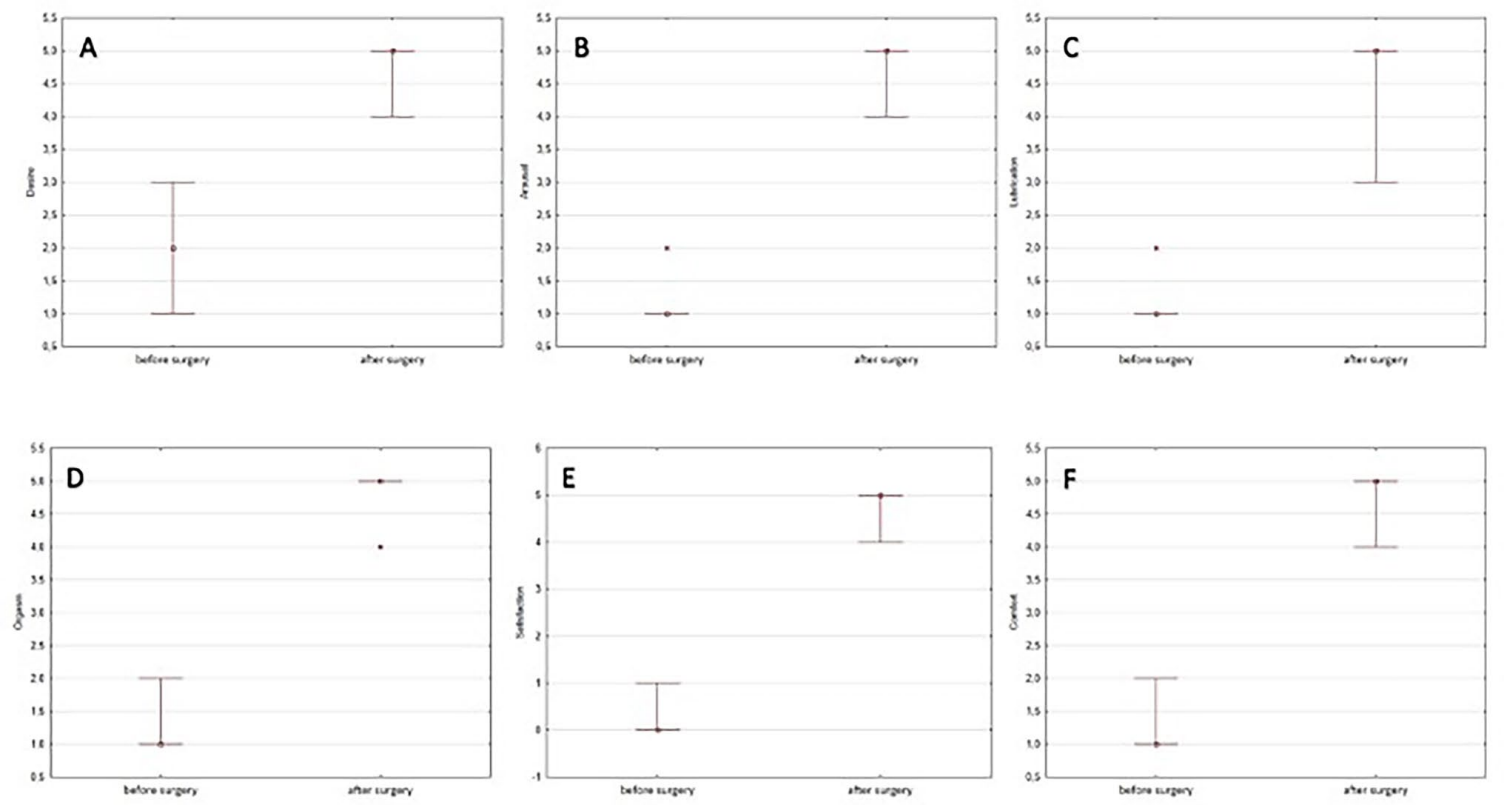
Female Sexual Function Index (FSFI) score in all six different domains: desire (panel **A**), arousal (panel **B**), lubrication (panel **C**), orgasm (panel **D**), satisfaction (panel **E**) and comfort (panel **F**) in women with Mayer-Rokitansky-Küster-Hauser syndrome before and after Davidov-Moore vaginoplasty

During follow-up, one woman had abnormal vaginal discharge without abdominal pain or fever which resolved after 5 days of oral treatment with antibiotics. No other complications such as bleeding, urine incontinence, or vaginal stenosis were recorded during the follow-up period. After a 6-month follow-up, vaginal length was 8.1-times longer than before surgery (Fig. 1, Table 1). The time from the surgery to the initiation of vaginal intercourse was between 17 to 22 weeks (women with perioperative internal bleeding). Mean FSFI score indicated good results, with no women below 23 score, and was 4.3- times higher when compared with pre-surgical one (Table 2). Among women with MRKH post-surgical FSFI revealed higher scores in all six different domains desire (2.5-times, Fig. 2 panel A), arousal (4.1-times, Fig. 2 panel B), lubrication (3.8-times, Fig. 2 panel C), orgasm (3.4-times, Fig. 2 panel D), satisfaction (3.3-times, Fig. 2 panel E) and comfort (11-times, Fig. 2 panel F) in contrary to initial FSFI before the surgery.

## Discussion

This study is the first to show that neovaginal creation with the Davidov-Moore approach is a safe treatment in women with MRKH. We also demonstrated the high effectiveness of the procedure reflected by prolonged vaginal length and good sexual function after this surgery.

Several studies have reported surgical and sexual outcomes after simple Davidov vaginoplasty [14]. All of them confirmed that the Davydov approach is a safe procedure, with operating time from 60 to 150 min [15], intraoperative blood loss of 50 to 10 ml [11], complication rate of 5 to 20% [16], and the hospital stay from 6 to 20 days [17, 18]. Except time of post-surgical healing, which was shorter in our study, we have observed comparable results. According to possible complications related to Davydov’s vaginoplasty, one study reported post-surgical urinary retention, requiring self-catheterization for 2 weeks along with stress incontinence recorded in one woman and urge incontinence in two women, anyhow, after 3-month follow-up, all three women were free of symptoms [19]. In our study, intraabdominal bleeding occurs in the youngest women with no other postoperative complications. Moreover, the sole Davydov vaginoplasty is not without long-term side effects. One study described bladder or rectum injuries, bladder-vaginal fistula along with vaginal dryness [12]. In our study, no bladder or rectum injuries or other complications occurred.

Laparoscopic Davidov vaginoplasty is reported to have favorable anatomic and sexual outcomes. Post-surgical outcomes revealed that women have more or less satisfactory sex lives, comparable to women without MRKH [15]. Few studies have shown that after at least 6 months from Davydov vaginoplasty, the mean post-surgical vaginal length was 8.3 cm (95% CI 8.1–8.6) which is comparable to our results which were achieved after the Davidov-Moore procedure [17, 20, 21]. Surprisingly, the neovaginal length of 3 months since Davidov surgery was up to 9.9 cm [18]. Evidently, sole vaginal length is not a fundamental determinant of women’s satisfactory sexual life [22]. One study reported that women with the neovaginal length of 4.5 cm, anyhow, married and with 1–2 per week of sexual intercourse, reported the highest FSFI score of 32.1 [15]. In the same study, Liu et al. [15] observed that a decrease in neovaginal length has dropped roughly along with the duration of wearing mold. In our study, the mean time of mold use, which was the same as initiation time, was nearly 4.9 months, whereas another study reported 6.1 months [17].

Sexual satisfaction in MRKH is frequently assessed using the FSFI score questionnaire [23]. Several studies have shown a total FSFI score above 28 after sole Davidov vaginoplasty which is in line with our Davidov-Moore results [17, 24]. However, in two studies total FSFI score was either higher – above 31 [25] or lower – around 26 [21]. One study hypothesized that shorter vaginal length, especially below 7 cm, might be associated with lower total FSFI score, anyhow, further studies are needed to elucidate this relationship.

The study has several limitations. Firstly, the sample size is limited and, therefore, our results should be interpreted with caution. Anyhow, further studies are needed to investigate possible surgery-related outcomes. Secondly, the follow-up period lasts only 6 months, thus, we are not able to exclude other long-term surgery-related complications. Finally, the significant associations showed in this study did not necessarily mean the cause-effect relationship.

In conclusion, laparoscopic Davydov-Moore vaginoplasty may be considered as a safe procedure with satisfactory anatomic and sexual outcomes, however, women need to be aware of possible complications. Further research is needed to investigate possible long-term outcomes and their clinical consequences.

## Data Availability

Data are available on request.
